# The mental health impact of the COVID-19 epidemic on hong kong youth: Preliminary results from the hong kong youth mental health epidemiological study (HKYES)

**DOI:** 10.1192/j.eurpsy.2021.744

**Published:** 2021-08-13

**Authors:** T.H. Chung, A.S. Yuen, C.S. Wong, C.L. Hui, S.K. Chan, W. Chang, E.H. Lee, E.Y. Chen

**Affiliations:** 1 Department Of Psychiatry, The University of Hong Kong, HK, Hong Kong PRC; 2 Department Of Psychiatry, The University of Hong Kong, Hong Kong, Hong Kong PRC

**Keywords:** youth, mental health, health anxiety, COVID-19

## Abstract

**Introduction:**

The 2019 coronavirus disease (COVID-19) is a global health crisis that originated in China. As an adjacent city to the origin of COVID-19, Hong Kong has been facing different public health challenges raised by the epidemic.

**Objectives:**

This paper examined the prevalence of common physical symptoms, psychological symptoms, somatic symptoms, and health anxiety among the Hong Kong youth population.

**Methods:**

HKYES is an on-going territory-wide epidemiological study collecting youth mental health data with randomly stratified sampling. Participants aged 15-24 years were to complete a physical symptom checklist, Depression, Anxiety and Stress Scale (DASS-21), Insomnia Severity Index (ISI), Patient Health Questionnaire-15 (PHQ-15), and Short Health Anxiety Inventory (SHAI).

**Results:**

A total of 594 participants have completed the survey since April 2020. The three most common physical symptoms were headache (n=106, 17.8%), fever (n=94, 15.8%) and fatigue (n=78, 13.1%). The mean scores of DASS depression, anxiety and stress subscales were 7.98 (SD 8.14), 5.81 (SD 6.32), and 8.83 (SD 7.93) respectively. Among all, 135 (22.8%) participants reported moderate to severe levels of depressive symptoms, 133 (22.4%) reported moderate to severe levels of anxiety symptoms, and 71 (12%) reported moderate to severe levels of stress. There were 40 (6.7%) and 60 (10.1%) participants showing significant levels of insomnia and somatic symptoms, while around one-third of the participants reported a high level of health anxiety.

**Conclusions:**

Youth is at risk of severe psychological impact during the coronavirus. Monitoring the mental health trajectory for youth should become routine practice during times of crisis.

